# Reversal of *IKZF1*-induced glucocorticoid resistance by dual targeting of AKT and ERK signaling pathways

**DOI:** 10.3389/fonc.2022.905665

**Published:** 2022-09-02

**Authors:** Miriam Butler, Britt M.T. Vervoort, Dorette S. van Ingen Schenau, Lieneke Jongeneel, Jordy C.G. van der Zwet, René Marke, Jules P.P. Meijerink, Blanca Scheijen, Laurens T. van der Meer, Frank N. van Leeuwen

**Affiliations:** ^1^ Princess Maxima Center for Pediatric Oncology, Utrecht, Netherlands; ^2^ Laboratory of Pediatric Oncology, Radboud Institute for Molecular Life Sciences, Radboud University Medical Center, Nijmegen, Netherlands; ^3^ Department of Pathology, Radboud University Medical Center, Nijmegen, Netherlands; ^4^ Radboud Institute for Molecular Life Sciences, Nijmegen, Netherlands

**Keywords:** leukemia, glucocorticoids, IKZF1, therapy resistance, AKT, ERK

## Abstract

Although long-term survival in pediatric acute lymphoblastic leukemia (ALL) currently exceeds 90%, some subgroups, defined by specific genomic aberrations, respond poorly to treatment. We previously reported that leukemias harboring deletions or mutations affecting the B-cell transcription factor *IKZF1* exhibit a tumor cell intrinsic resistance to glucocorticoids (GCs), one of the cornerstone drugs used in the treatment of ALL. Here, we identified increased activation of both AKT and ERK signaling pathways as drivers of GC resistance in *IKZF1-*deficient leukemic cells. Indeed, combined pharmacological inhibition of AKT and ERK signaling effectively reversed GC resistance in *IKZF1*-deficient leukemias. As inhibitors for both pathways are under clinical investigation, their combined use may enhance the efficacy of prednisolone-based therapy in this high-risk patient group.

## Introduction

Despite improvements in up-front treatment regimens and the introduction of immunotherapies, about 10-15% of the pediatric acute lymphoblastic leukemia (ALL) patients develop relapse disease, which remains difficult to treat and almost half of the patients will ultimately succumb to their disease (1–3).

Several genetic aberrations are associated with an increased relapse risk. Deletions or mutations affecting the B-cell transcription factor *IKZF1*, which occur in 10-15% of pediatric B-cell progenitor ALL (BCP-ALL), were shown to negatively affect cellular responses to a number of therapeutic drugs, thereby increasing the chance of therapy failure (4, 5). Alterations in *IKZF1* induce therapy resistance of leukemic cells *via* distinct mechanisms, including enhanced integrin-dependent survival signaling through activation of focal adhesion kinase (FAK) (6) and increasing cell intrinsic resistance to glucocorticoids (GCs) (7–9).

Synthetic glucocorticoids are a central component of the multi-drug treatment protocols of ALL worldwide. These drugs act by binding to the glucocorticoid receptor (GR), a ligand activated transcription factor located in the cytosol, which translocates to the nucleus to regulate transcription of GR-target genes. Resistance to GCs is recognized as one of the main causes of relapse in ALL (10), and as a result, the initial response to prednisolone is a strong predictor of treatment outcome (11–13). There are several mechanisms that contribute to GC resistance, such as reduced expression or loss of function of the GR, alterations in cellular metabolism, or resistance to apoptosis (e.g. *via* Bcl2 family members), but restoring GC therapy response has remained challenging (10). We previously showed that IKZF1 alterations confer resistance to GC treatment by inhibition of GC-induced transcriptional responses, but the exact molecular mechanisms underlying these effects remain to be elucidated (7).

Recently, *IKZF1* was identified as transcriptional repressor of two PI3K pathway related genes (PIK3CD and PIKFYVE) in T-ALL (14), leading to the induction of AKT signaling in response to *IKZF1* loss. In T-ALL, AKT1 activity was shown to be a determinant of GC resistance, driving phosphorylation of the GR to prevent its nuclear translocation and, consequently, glucocorticoid-induced gene expression (15). Indeed, pharmacological inhibition of AKT effectively reversed GC resistance both *in vitro* and *in vivo* (15). Also in BCP-ALL there is evidence that AKT signaling can induce GC resistance, as components of the AKT pathway appear to be over-activated in GC-resistant pre-B-ALL samples (16). Moreover, it was shown that CRLF2 rearranged leukemias can be re-sensitized to GCs by targeting of AKT (17). Here, we explored the connection between loss of IKZF1 function, AKT signaling and GC therapy resistance using an isogenic cell line model, normal B cells derived from *Ikzf1* ^+/-^ mice as well patient-derived ALL xenografts.

## Methods

### Ethical statement

Patient derived xenografts were generated from patient samples collected from different countries within the International BFM Study Group (I-BFM-SG) and the Dutch Childhood Oncology Group. All patients were enrolled in trials on treatment of pediatric ALL conducted by individual member groups of the I-BFM-SG: the AIEOP-BFM study group (Austria, Germany, Italy and Switzerland), the FRALLE study group (France) and the United Kingdom (UK) National Cancer Research Institute (NCRI) Childhood Cancer and Leukemia Group and DOCG Group. All treatment trials were approved by the respective national institutional review boards, and informed consent for the use of spare specimens for research was obtained from study individuals, parents or legal guardians.

### Plasmids

The following plasmids were obtained *via* Addgene:

pS-Pax2 (#12260), pMD2.G (#12259), pL-CRISPR.EFS.GFP (#57818), pLKO5.sgRNA.EFS.tRFP (#57823). For targeted knockout, gRNA sequences (**Supplemental Table 1**) were cloned into pL-CRISPR.EFS.GFP and pLKO5.sgRNA.EFS.tRFP using the BsmBI sites and the sequence integrity was verified using Sanger sequencing.

### Reagents

Prednisolone, MK2206, Uprosertib and SCH772984 were purchased from Selleckchem (Munich, Germany) and dissolved as instructed by the manufacturer.

### Cell culture

SEM cells (ACC 546) were obtained from the Leibniz Institute DSMZ (German Collection of Microorganisms and Cell Cultures, Braunschweig, Germany) and maintained in RPMI-1640 medium (Invitrogen, Thermo Fisher Scientific, Breda, the Netherlands), supplemented with 10% fetal bovine serum (FBS, Greiner Bio-One, Essen, Germany) and 1% penicillin/streptomycin solution (P/S) (Invitrogen) at a cell density between 0.25 and 3x10^6^ cells per milliliter.

Hek293FT cells were purchased from Invitrogen and maintained in DMEM, supplemented with 10% FBS, 1% non-essential amino acids (Gibco, Thermo Fischer) and 1% P/S. Cell cultures were tested regularly for the presence of mycoplasm. Cell line identity was confirmed by DNA fingerprinting.

### Lentivirus production and transduction

HEK293FT packaging cells (Invitrogen) were transfected with a viral backbone encoding the sgRNAs or Cas9 and the helper plasmids for virus production (psPAX2 and pMD2.G) using Polyethylenimine (PEI). Virus containing supernatant was collected 2 days after transfection. Virus was concentrated by centrifugation at 25,000G for 1 hour at 4°C and resuspended in the cell culture medium required by the target cells. Target cells (0.5-1x10^6^) cells were transduced with 1 ml virus (1-10 times concentrated) using spinoculation for 45 min at 700g (30°C) in the presence of 5 ug/ml polybrene (Santa Cruz Biotechnology, Dallas, TX). 72-96 hours after transduction GFP/RFP positive cells were sorted as single cell per well by flow cytometry using a H800S Cell Sorter (Sony Biotechnology). Single cell clones were individually analyzed for evidence of mutations and IKZF1 expression.

### Sanger sequencing

Genomic DNA was isolated using the Nucleospin genomic DNA isolation kit (#740952, Machery-Nagel, Bioké, Leiden, the Netherlands). The *IKZF1* exon 3 locus was amplified by PCR and cloned into the pGEM-t-easy plasmid (Promega, Madison, Wi) and transformed to *E.coli*. Plasmid DNA was isolated from single colonies using the Nucleospin easypure kit (#740727, Machery-Nagel) and at least 10 colonies per genotype submitted for Sanger sequencing (Macrogen, Amsterdam, the Netherlands) to ensure sufficient coverage of both alleles.

### RNA sequencing

RNA sequencing and data analysis was performed by NovoGene (Cambridge, UK) on triplicate samples. mRNA samples from SEM WT and IKZF1^-/-^ cells were purified using a RNeasy minikit (#74106, Qiagen, Hilden, Germany) with on column DNAse (#79254, Qiagen) treatment. Aberrant splicing was visualized using the Integrative Genomics Viewer (IGV, Broad Institute, Cambridge, MA).

### Cell viability assays

Cell viability was determined by flow cytometry using amine staining to discriminate between live and dead cells. Cells were seeded in a 96-well plate, in a 24-well plate or in a 6-well plate at 500,000 cells per ml. After the indicated incubation times, cells were stained with LIVE/DEAD™ Fixable Dead Cell Stain Sampler Kit (Thermo-Fischer, L34960) according to the manufacturer’s instructions and analyzed by Fluorescence Activated Cell Sorting (FACS) using a LSRII flow cytometer (BD Biosciences, Breda, The Netherlands) or CytoFLEX LX (Beckman Coulter). For co-culture experiments, MSCs were gated out *via* FSC/SSC gating. The data were collected and analyzed using FlowJo V10 software (FlowJo, Ashland, Oregon).

### *Ex vivo* culture of patient-derived xenografts

Patient-derived xenografts (PDXs) were generated as described before by intra-femoral injection of 1 × 10^5^ to 5 × 10^6^ viable primary ALL cells in NOD.Cg-Prkdc^scid^Il2rg^tm1Wjl^/SzJ (NSG) mice (18). The *ex vivo* co-culture method has been described previously (19). In short, hTERT immortalized MSCs (20) were seeded in a 96-wells format (14,000 cells/well) 24 hours prior to the addition of ALL xenografts (140,000 cells/well). ALL cells were allowed to settle for 24 hours before drugs were added. After 3d of drug incubation, cells were stained with LIVE/DEAD™ Fixable Dead Cell Stain Sampler Kit (Thermo-Fischer, L34960) as described previously. Cytogenetic properties of all PDX can be found in **Supplemental Table 2**.

### Experimental procedures Ikzf1 knockout animals

The Ikzf1Neo knockout mouse line that was used in this study was kindly provided by M. Busslinger (Research Institute of Molecular Pathology, Vienna, Austria). The Ikzf1Neo strain was maintained as a heterozygous knockout line (Ikzf1^+/-^) on an inbred C57BL/6J genetic background, and all animals were housed under specific pathogen-free conditions. All animal experiments were licensed by the Dutch Central Authority for Scientific Procedures on Animals and approved by the Animal Welfare Body of the Radboud university medical center and performed in accordance with institutional and national guidelines. Wild-type and Ikzf1^+/-^ mice were sacrificed at the age of 8 to 14 weeks after which spleens were removed. Single cell suspensions of splenocytes were obtained using a 70 μM cell strainer. Erythrocytes in the isolates were removed by lysis in red blood cell lysis buffer (Sigma-Aldrich, Zwijndrecht, Netherlands). Single cell splenocytes were cultured for 48 hours in RPMI-1640 medium (Life technologies, Carlsbad, CA) supplemented with 10% heat-inactivated fetal calf serum (FCS), 1% penicillin/streptomycin (Invitrogen), and 50 μM β-Mercaptoethanol in the presence of 5 μg/ml lipopolysaccharide (LPS). Stimulated and viable splenic B-lymphocytes obtained after Ficoll gradient purification were subsequently cultured in a 96-well plate at a density of 1x 105 cells/well under similar conditions as in the initial expansion phase. B cells were treated with indicated doses of drugs. After 72 hours, cells were prepared for immunoblotting as stated below.

### Real-time quantitative polymerase chain reaction

Total RNA was extracted using a RNeasy mini-kit (Qiagen). Subsequently, cDNA was synthesized of 500ng RNA template using the iScript cDNA synthesis kit (Bio-Rad, Hercules, CA). mRNA expression levels were determined using a Power SYBR^®^ Green PCR master mix with gene-specific primers (sequences are listed in **Supplementary Table 1**) and a CFX96 Touch Real-Time PCR detection system (Bio-Rad, Hercules, CA, USA). HPRT mRNA expression was employed as a reference to obtain the fold change in expression levels of target genes using the comparative cycle threshold 2(-ΔΔCt) method. Primer sequences are listed in **Supplemental Table 1**.

### Immunoblotting

Cells were lysed in Laemmli protein buffer and treated with benzonase nuclease (Sigma Aldrich) for 30 minutes, prior to boiling. Purified proteins were separated by SDS-PAGE and transferred to PVDF membranes or nitrocellulose membranes (Amersham Biosciences). After protein transfer, membranes were blocked in TBS-5% elk and incubated with primary antibodies (Staining conditions can be found in **Supplemental Table 3**), washed in TBS-0.02% Tween, followed by incubation with an IRDye conjugated secondary antibody (Li-cor, Biotechnology). Proteins were visualized and quantified with the Odyssey^®^ CLx and accompanying Image Studio software (Li-cor, Biotechnology).

### Statistical analyses

Statistical analyses for the cell viability, quantified protein expression and qRT-PCR assays were performed using PRISM6 (GraphPad Software, La Jolla, CA). For most dose-response curves, the shape of the curve did not allow curve fitting and IC50 calculations. Therefore, we calculated the area under the curve and tested differences for significance using a one-way ANOVA and Tukey’s multiple comparisons test. For Western blot quantifications and qRT-PCR a two-sided student’s t test and one-way ANOVA followed by Dunnett’s multiple comparisons test were performed. *p*-values < 0.05 were considered statistically significant (*p<0.05, **p<0.01, ***p<0.001, ****p<0.0001).

## Results

### IKZF1 regulates AKT signaling

**Figure 1:**
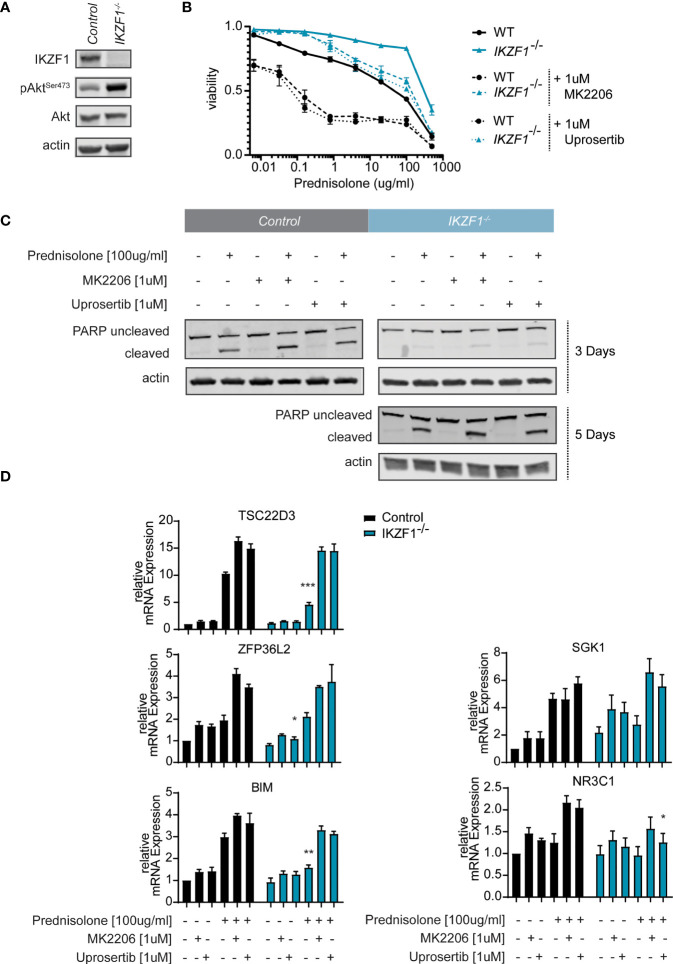
**(A)** Immunoblot analysis of protein expression in SEM IKZF1 knockout cells generated by CRISPR/Cas9-based targeting of the *IKZF1 locus*. Representative blot of three independent experiments. Protein expression was quantified, normalized and tested for differences using a student’s t-test. p-values are plotted in **Supplemental Figure 2A**. **(B)** Cell death as determined by quantification of cells positive for amine-reactive dyes using flow cytometry in SEM wt and SEM *IKZF1^-/-^* cells after a 4-day treatment with increasing concentrations of prednisolone in the presence or absence of 1 μM MK2206 or uprosertib. Each data point represents a mean (± Standard Error of the Mean (SEM)) of 3 independent experiments. The area under the curve (AUC) was determined and differences were tested for significance using an ANOVA followed by Tukey’s multiple comparisons test. P-values of the comparisons are plotted in **Supplemental Table 4**. **(C)** Immunoblot analysis of protein expression in SEM wt and SEM *IKZF1* ^-/-^cells after a 3 day and 5-day (for the knockout cells) treatment or 100 μg/ml prednisolone, 1 μM MK2206 or uprosertib or the combination of prednisolone with the AKT inhibitor. Representative blot of three independent experiments. Protein expression was quantified, normalized and tested for differences using an ANOVA followed by Dunnett’s multiple comparisons test. p-values are plotted in **Supplemental Figure 2B**. **(D)** mRNA expression analysis of GR-target genes TSC22D3, ZFP36L2 and SHK1, the pro-apoptotic protein BIM and the glucocorticoid receptor NR3C1 was performed on RNA isolated SEM wt and SEM *IKZF1^-/-^* cells after a 16 hours treatment of 100 μg/ml prednisolone, 1 μM MK2206 or uprosertib or the combination of prednisolone with the AKT inhibitor. Each bar represents a mean (± SEM) of 3 independent experiments. All values were normalized to their untreated control. Differences in expression between the genotypes and between the treatment conditions were tested for significance using either a student’s t-test or ANOVA followed by Dunnett’s multiple comparisons test. p-values are listed in **Supplemental Table 5**. *p < 0.05; **p < 0.01; ***P < 0.001.

CRISPR/Cas9-based genome editing was used to target the *IKZF1* locus in SEM pro-B-ALL cells. Since we found a strong selection against cells carrying a heterozygous loss of IKZF1, leading to an instable phenotype (not shown), we used a homozygous knockout model to assess the effects of IKZF1 loss (**Figure 1A** and **Supplemental Figures 1A, B**). Similar to what has been observed in primary patient samples, disrupted IKZF1 expression led to increased resistance against prednisolone treatment. Analogous to what has been observed in T-ALL (14), a loss of IKZF1 expression was accompanied by enhanced phosphorylation of AKT, while total AKT protein expression remained unaffected (**Figure 1A** and **Supplemental Figure 2A**). To demonstrate that the effect on AKT phosphorylation was a direct consequence of *IKZF1* loss, and independent of the leukemic context, we investigated AKT status in LPS-activated B cells from *Ikzf1^+/-^* mice (**Supplementary Figures 3A, B**). Previously, we showed that these cells are about 100-fold more resistant to synthetic GCs compared to B cells derived from *Ikzf1* wild-type mice (7). Indeed, similar to IKZF1^-/-^ SEM cells, enhanced AKT phosphorylation was observed in *Ikzf1^+/-^* B cells as compared to wild type B cells isolated from control (**Supplementary Figure 3B**).

### Inhibition of AKT signaling partly restores GC-sensitivity in IKZF1^-/-^ BCP-ALL cells

To test whether AKT functions as a mediator of GC resistance in *IKZF1*-deficient BCP-ALL cells, we treated control and *IKZF1^-/-^* cells with increasing concentrations of prednisolone in the presence or absence of AKT inhibitor MK2206 (21). Indeed, silencing of AKT activity partially re-sensitized *IKZF1^-/-^* cells to prednisolone treatment (**Figure 1B** and **Supplemental Table 4**). We confirmed our observations using uprosertib (GSK2141795) (22), a structurally unrelated AKT inhibitor which is under clinical evaluation for a variety of cancer types (23, 24). To confirm that AKT inhibition enhanced the prednisolone induced cell killing we used Western blot to test for PARP cleavage on protein extracts from cells exposed to prednisolone in the presence or absence of AKT inhibitors. Similar to what we observed using the amine staining, the addition of either inhibitor led to an increase apoptosis in both wildtype and knockout cells. Although the wildtype cells responded faster to treatment, longer exposure to the drugs resulted in an increased PARP cleavage in the IKZF1 deficient cells, which was further substantiated by AKT inhibition by MK2206 or Uprosertib (**Figure 1C** and **Supplemental Figure 2B**).

To further explore the effects of AKT inhibition on GC-dependent target gene regulation, quantitative reverse transcription PCR (qRT-PCR) was performed on a subset of established GC response genes, including *TSC22D3, ZFP36L2 and SHK1 (* 7). Incubation of control and IKZF1^-/-^ cells with uprosertib enhanced the expression levels of all these response genes in wild-type SEM cells (**Figure 1D** and **Supplemental Table 5**). More importantly, we observed that the attenuated transcriptional response to prednisolone, that we observed in SEM *IKZF1^-/-^* cells, was restored by AKT inhibition (**Figure 1D**). Of note, similar to what has been described before (7), we found no evidence that basal expression of the glucocorticoid receptor NR3C1 was affected by loss of IKZF1.

As induction of apoptosis in response to GCs is dependent on a cell’s capacity to induce BIM expression (25, 26), we analyzed Bim mRNA expression in response to treatment with prednisolone, AKT inhibition or the combination. Indeed, prednisolone-mediated induction of Bim mRNA expression was reduced in *IKZF1^-/-^* cells relative to control cells, but could be rescued by treatment with AKT inhibitors (**Figure 1D** and **Supplemental Table 5**). We conclude from these results that GC-resistance caused by *IKZF1* loss can, at least in part, be attributed to upregulation of AKT signaling, a process that can be reversed by pharmacological targeting of AKT. Given the fact that the two distinct AKT inhibitors performed highly similarly in restoring the sensitivity to prednisolone, we continued our experiments using MK2206, which is more widely tested in clinical studies.

### Combined inhibition of AKT and ERK restores GC-sensitivity in IKZF1-deficient ALL cell models

**Figure 2:**
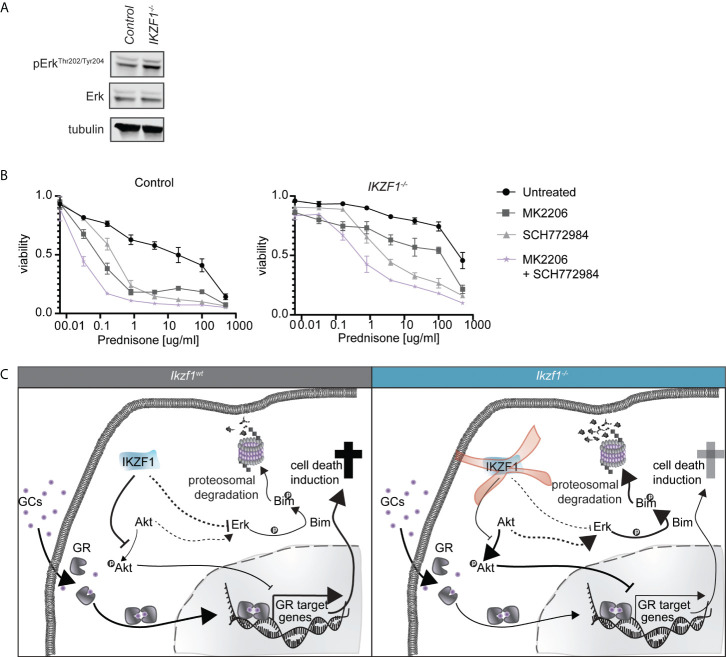
**(A)** Immunoblot analysis of protein expression in IKZF1 wildtype and deficient SEM cells. Phospho-ERK expression was quantified, normalized to total ERK expression and tested for differences using a student’s t-test (**Supplemental Figure 4**). **(B)** Cell death as determined by quantification of cells positive for amine-reactive dyes using flow cytometry in SEM wt and SEM *IKZF1^-/-^* cells after a 4-day treatment with increasing concentrations of prednisolone in the presence or absence of 1 μM MK2206, SCH772984 or their combination. Each data point represents a mean (± SEM) of 3 independent experiments. The area under the curve (AUC) was determined and differences were tested for significance using an ANOVA followed by Tukey’s multiple comparisons test. p-values of the comparisons are listed in **Supplemental Table 6**. **(C)** Schematic representation of working model explaining how loss of *IKZF1* might induce prednisolone resistance. In *IKZF1* ^wt^ cells, IKZF1 represses AKT activity. Upon loss of *IKZF1*, AKT activity is no longer suppressed. This results in repression of GR target gene activation upon prednisolone treatment. At the same time, ERK is activated either *via* crosstalk with AKT signaling or directly *via* IKZF1 which, in turn, can phosphorylate Bim inducing proteasomal degradation.

Previous studies suggest that inhibition of MAPK/ERK signaling enhances GC-mediated cell killing in both B- and T-ALL cells (27, 28). For this reason, a combination of the MEK inhibitor selumetinib and dexamethasone is now under clinical investigation for the treatment of ALL (NCT03705507). Moreover, from a wide range of human cancers we know that there is extensive cross-talk between AKT and MAPK/ERK signaling (29, 30), we observed that inhibition of AKT alone resulted in partial suppression of ERK phosphorylation in both control and *IKZF1^-/-^* cells (**Supplementary Figure 3C**). In T-ALL dual targeting has been shown to act synergistically with prednisolone (31). Given the important role of MAPK/ERK signaling in regulating GC therapy response (27, 28), we investigated whether loss of IKZF1 affected ERK activation. Indeed, phosphorylation of ERK appeared to be elevated in *IKZF1* ^-/-^ cells relative to wildtype cells (**Figure 2A** and **Supplemental Figure 4**).

To assess consequences for prednisolone induced apoptosis, we treated control and *IKZF1* ^-/-^ cells with increasing concentrations of prednisolone in the presence or absence of AKT inhibitor MK2206, the ERK inhibitor SCH772984 or the combination thereof. Inhibition of either AKT or ERK alone enhanced prednisolone induced apoptosis in both control and *IKZF1* ^-/-^ cells, but this effect was strongly potentiated when both inhibitors were combined (**Figure 2B** and **Supplemental Table 6**).

Together these findings support a model in which loss of *IKZF1* promotes activation of AKT as well as MAPK/ERK signaling, the latter one either independent or mediated through AKT signaling, both of which contribute to resistance to GCs in response to *IKZF1* loss. More importantly, combined targeting of AKT and ERK restores GC-induced apoptosis in *IKZF1* ^-/-^ cells (**Figure 2C**).

### Dual targeting of AKT and ERK signaling reverses prednisolone resistance in patient-derived xenografts in response to the IKZF1-degrading drug iberdomide

Immunomodulatory agents (IMiDs) such as lenalidomide, pomalidomide and iberdomide show efficacy in the treatment of myeloid malignancies, including acute myeloid leukemia (AML), multiple myeloma and myelodysplastic syndromes (MDS). Although the exact mechanism behind the anti-tumor activity remains to be established, these compounds were found to act both on the microenvironment as well as on the immune system (32, 33). On a cellular level, these compounds act as potent modulators of the Cereblon E3 ubiquitin ligase complex, and were found to redirect the E3 ubiquitin ligase complex to specific cellular targets, particularly IKZF1 and IKZF3 (34). This mechanism of action is currently exploited in the development of many other compounds to target ‘undruggable’ proteins for degradation in a new class of drugs known as proteolysis targeting chimera (PROTAC). We explored the effects of iberdomide-mediated modulation of IKZF1 protein levels on therapy responses in primary patient cells that were previously expanded using xenotransplantation in immunodeficient mice (PDX) wild type for *IKZF1*. This allowed us to compare the effects of IKZF1 loss in primary patient cells that are hard to genetically manipulate as they cannot be cultured *ex vivo*.

**Figure 3:**
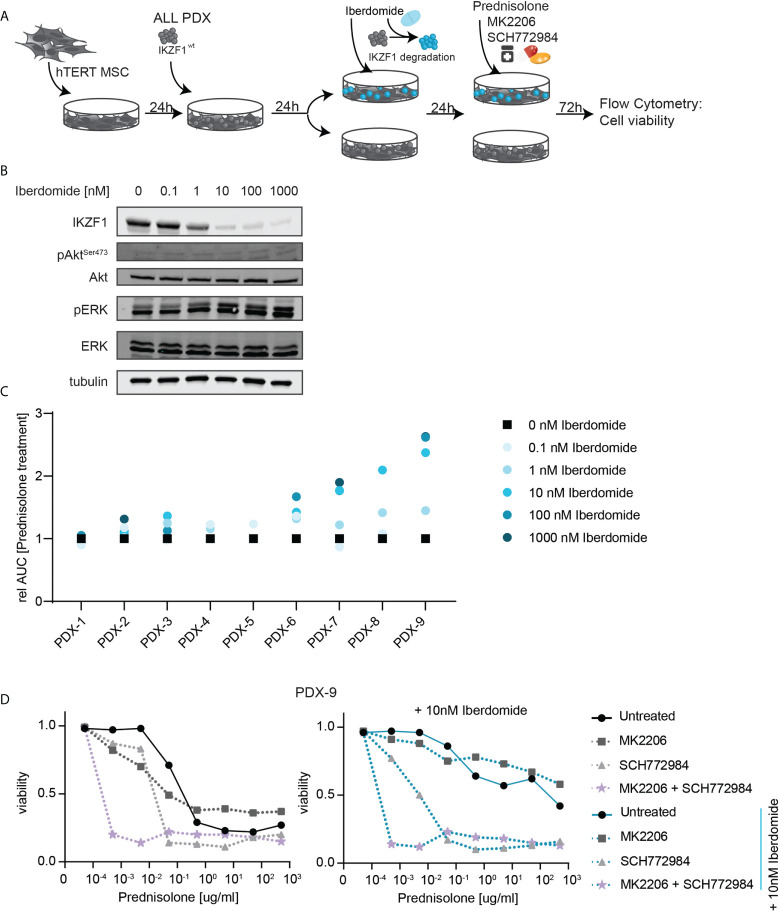
**(A)** Schematic overview representing the workflow used to determine *ex-vivo* prednisolone responses in PDX samples upon iberdomide treatment. ALL PDX samples wild type for *IKZF1* were grown on a feeder layer of hTERT immortalized mesenchymal stem cells (MSCs) and left untreated or exposed to the IKZF1 degrading drug Iberdomide. Then cells were treated with increasing concentrations of prednisolone in the presence or absence of MK2206, SCH772984 or their combination. After 3 days of incubation cell death was determined by quantification of cells positive for amine-reactive dyes using flow cytometry. **(B)** Immunoblot analysis of IKZF1, pAKT and pERK protein expression in a representative PDX sample wildtype for IKZF1 in response to iberdomide (24h). **(C)** Relative sensitivity to prednisolone of iberdomide treated cells. PDXs wt for IKZF1 were treated with indicated concentrations iberdomide after which responses to prednisolone were determined by quantification of cells positive for amine-reactive dyes using flow cytometry. The AUC of iberdomide treated cells was divided by the AUC of control treated cells to determine changes in sensitivity to prednisolone following iberdomide treatment. **(D)** Prednisolone induced cell death as determined by quantification of cells positive for amine-reactive dyes using flow cytometry in PDX-9. Cells were either pre-treated with 10 nM Iberdomide or left untreated for 24h before they were treated for 3-days with increasing concentrations of prednisolone in combination with MK2206 and/or SCH772984.

PDX samples wildtype for *IKZF1* were exposed *ex vivo* to different doses of iberdomide for 24 hours, prior to analyzing their response to prednisolone. Indeed, we observed that treatment with iberdomide led to a downregulation of IKZF1 protein expression (**Figures 3A, B**). Although we could not detect pAKT in PDX samples, iberdomide treatment resulted in a measurable increase in ERK phosphorylation (**Figure 3B**).

More importantly, exposure to iberdomide phenocopied the effects of genomic loss of *IKZF1* by inducing resistance to prednisolone in 8 out of 9 PDXs wildtype for *IKZF1* (**Figure 3C**).

To assess consequences for prednisolone induced apoptosis, we treated control and Iberdomide treated cells of PDX-9, the sample in which iberdomide treatment induced the strongest prednisolone resistance, with increasing concentrations of prednisolone in the presence or absence of the AKT inhibitor MK2206, ERK inhibitor SCH772984 or a combination thereof. Notably, AKT inhibition as a single agent produced little effect on prednisolone-induced apoptosis, whereas ERK inhibition strongly synergized with prednisolone treatment in both iberdomide-treated and non-treated cells. Combining ERK and AKT inhibition, these cytotoxic effects were strongly enhanced. Even at the lowest prednisolone concentration (0.0005ug/ml), reduced cell viability was observed in both control and iberdomide treated samples when AKT and ERK signaling pathways were inhibited (**Figure 3D**). Notable exceptions were t(1;19) (TCF3-PBX1) rearranged leukemias, in which iberdomide-induced *IKZF1* loss led to opposite effects, causing resistance to GCs (**Supplementary Figure 5**). Consistent with this observation, AKT inhibition rendered t(1;19) positive PDXs more resistant to GC-therapy (data not shown). Although this discrepancy awaits further explanation, it points to a unique biology for t(1;19)-positive ALL.

### IKZF1-deficient ALL can be re-sensitized to prednisolone treatment by combined AKT/ERK inhibition

**Figure 4:**
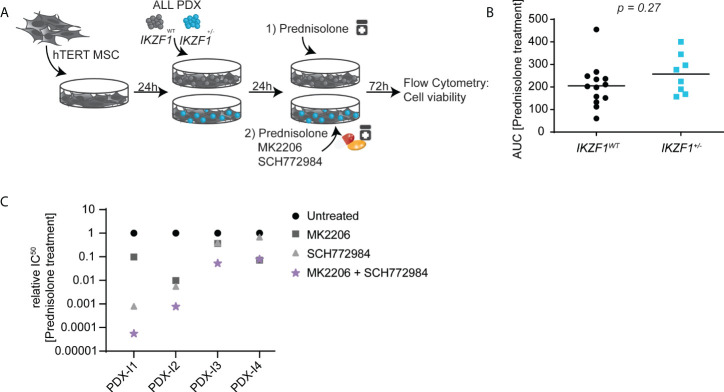
**(A)** Schematic overview representing the workflow used for ex-vivo co-cultures. In short, hTERT MSCs were seeded in 96-wells plate and allowed to settle for 24h before ALL-PDXs either wt or with a hemizygous *IKZF1* deletion were added and again allowed to settle for 24h. Then, cells were incubated with different drug concentrations and after 3 days incubation, cell death was determined by using an amine-staining using Flow cytometry. **(B)** Cell viability determined by amine staining in patient-derived xenografts either wild type (black) or carrying a heterozygous deletion (blue) of *IKZF1* depicted as Area Under the Curve. **(C)** Prednisolone induced cell death as determined by quantification of cells positive for amine-reactive dyes using flow cytometry in ALL-PDXs carrying a heterozygous deletion of *IKZF1*. Cells were treated for 3-days with increasing concentrations of prednisolone in combination with MK2206 and/or SCH772984 after which IC50 values were calculated and plotted relative to prednisolone-only treated cells. The corresponding dose-response graphs are shown in **Supplemental Figure 6**. For PDX-I5, IC50 calculation was not possible and only the dose response is shown in **Supplemental Figure 6**.

Next, we investigated to what extent either AKT inhibition or ERK inhibition, and a combination thereof, could increase the *ex vivo* sensitivity for prednisolone in BCP-ALL PDXs harboring an endogenous *IKZF1* gene deletion (**Figure 4A**). First, the prednisolone response was tested in a panel of 21 ALL PDXs, derived from samples obtained at diagnosis, that were either wildtype for *IKZF1* (n=13) or *IKZF1*-deficient (n=8). Cells were incubated with increasing concentrations of prednisolone and after 3 days, cell viability was measured by staining with amine reactive dyes and quantified by flow cytometry. Data were plotted as dose-response curves and the area under the curve (AUC) was calculated as measure for sensitivity. In accordance with previous published data (7) *IKZF1*-deficient PDXs on average were more resistant to prednisolone compared to PDXs wild type for *IKZF1*, albeit with a large variation in sensitivity (**Figure 4B**).

Upon inhibition of either AKT or ERK signaling, prednisolone-induced apoptosis was generally potentiated in *IKZF1*-deficient PDXs (**Figure 4C** and **Supplementary Figure 6**). However, the extend was highly variable between PDX samples and in general inhibition of ERK signaling was more effective in restoring GC sensitivity as compared to that of AKT inhibition, but also more active as single agent. However, in all *IKZF1*-deficient PDXs, a combination of both inhibitors strongly increased prednisolone-induced apoptosis, again highlighting the potential of combined AKT and ERK inhibition as a therapeutic option to re-sensitize this high-risk patient group to prednisolone treatment.

## Discussion

In this study we showed that IKZF1-mediated regulation of AKT and ERK signaling contributes to GC resistance in BCP-ALL. Loss of IKZF1 function leads to activation of AKT as well as MAPK/ERK signaling, resulting in GC treatment resistance. Combined inhibition of MAPK/ERK and AKT signaling restores GC-induced apoptosis in *IKZF1^+/-^* cells. This sensitizing effect is observed in BCP-ALL cell lines and PDXs. Similar to T-ALL (14), IKZF1 may act to suppress AKT signaling in BCP-ALL leading to increased AKT signaling in response to *IKZF1* loss, thereby inducing GC resistance by repression of GC-induced transcriptional responses. At the same time, ERK phosphorylation appears to be induced, either by crosstalk with AKT signaling or another, yet unknown IKZF1-mediated mechanism. Inhibition of both pathways may represent a potential Achilles’ heel for GC resistance in IKZF1 deficient tumors. The availability of clinical grade small molecule inhibitors for both pathways would in principle allow a rapid translation to the clinic (35, 36). The challenge will be to combine these agents at clinically effective doses while mitigating toxicities associated with these drugs, such as rash and diarrhea (37, 38).

Another challenge remaining is to distinguish responders from non-responders. Although in the majority of *IKZF1* deficient PDXs, prednisolone-induced apoptosis was potentiated by either AKT inhibition, ERK inhibition or their combination, the extent of synergy varied between patient samples. This might partially be explained by other co-occurring genetic lesions as the effects *IKZF1* deletions on outcome are dependent on other co-occurring genetic events (39). For instance, *BTG1* deletions (40) or the so called *IKZF1^plus^* group (*IKZF1* deletions co-occurring with deletions in *CDKN2A, CDKN2B, PAX5, or PAR1* in the absence of *ERG* deletions) (41) enhance the negative impact of *IKZF1* deletions on outcome, whereas *ERG* deletions abrogate this effect (42). However, in our small sample set, the *IKZF1* ^plus^ group (PDX-I4, PDX-I5, PDX-I3, PDX-I2) displayed no correlation with response to AKT/ERK inhibition. Interestingly, the only PDX completely unresponsive to AKT inhibition (PDX-I5, **Supplemental Figure 6**) was a BCR-ABL1 positive leukemia that also harbored a *TP53* deletion. This tumor suppressor is known to affect cancer metabolism and, consequently, the way tumor cells respond to treatment (43–46), which may explain its unresponsiveness to AKT inhibition. The fact that most, but not all tumors respond to this combination treatment suggest that a personalized medicine approach, possibly guided by *ex-vivo* drug testing of primary patient samples may be used to identify and treat patients that may benefit from this treatment.

In summary our study identified increased activation of both AKT and ERK signaling pathways as drivers of GC resistance in IKZF1-deficient leukemic cells, while by combined pharmacological inhibition of both pathways GC resistance could be effectively reversed. As inhibitors for both pathways are under clinical investigation, their combined use may enhance the efficacy of prednisolone in this high-risk patient group.

## Data availability statement

Requests to access the datasets should be directed to FN.vanLeeuwen@prinsesmaximacentrum.nl.

## Ethics statement

The studies involving human participants were reviewed and approved by I-BFM-SG: The AIEOP-BFM study group (Austria, Germany, Italy and Switzerland), the FRALLE study group (France) and the United Kingdom (UK) National Cancer Research Institute (NCRI) Childhood Cancer and Leukemia Group and DOCG Group. Written informed consent to participate in this study was provided by the participants’ legal guardian/next of kin.

The animal study was reviewed and approved by Dutch Central Authority for Scientific Procedures on Animals and the Animal Welfare Body of the Radboud university medical center.

## Author contributions

FvL, LvdM, and BS conceptualized the project and supervised the work. MB and BV performed the experiments with assistance from RM, DvIS, JvdZ, and LJ. DvIS generated the xenografts, JM supervised JvdZ. MB, LvdM, and FvL drafted the manuscript which was edited, reviewed and approved by all authors.

## Funding

This work was supported in part by research funding from the Dutch Cancer Society (KWF) (grant #10072 and #11249) and from KiKa (grant #333), and a young investigator grant from Radboudumc (MB).

## Acknowledgments

We are thankful to Didier Trono for providing psPAX2 (Addgene plasmid # 12260) and pMD2.G (# 12259) plasmids and to Benjamin Ebert for sharing the pL-CRISPR.EFS.GFP (#57818) and pLKO5.sgRNA.EFS.tRFP (#57823) plasmids. In addition, we want to thank the members of the PRIME department of the Radboud umc animal facility for valuable technical support.

## Conflict of interest

The authors declare that the research was conducted in the absence of any commercial or financial relationships that could be construed as a potential conflict of interest.

## Publisher’s note

All claims expressed in this article are solely those of the authors and do not necessarily represent those of their affiliated organizations, or those of the publisher, the editors and the reviewers. Any product that may be evaluated in this article, or claim that may be made by its manufacturer, is not guaranteed or endorsed by the publisher.

## Supplementary material

Supplementary Figure 1
**(A)** CRISPR/Cas9 was used to target the exon 3 locus of the *IKZF1* gene in the SEM cells. After clonal expansion, the genomic DNA was analyzed by Sanger sequencing, revealing two insertions: c.del 51_86 CCCCCCTGTAAGCGATACTCC-AGATGAGGGCGATGA and c.del 149_154 ACAGAG **(B)** Analysis of mRNA sequencing data revealed that the genomic deletions resulted aberrant mRNA mRNA splicing and as a consequence, absence of detectable protein ().

Supplementary Figure 2
**(A)** Quantification of phospho-AKT, normalized for total AKT protein expression in SEM cells wildtype or deficient for IKZF1. The western blot results for 3 independent experiments were quantified, normalized and plotted. Differences were tested for significance using a student’s t-test **(B)** Quantification of cleaved PARP protein, normalized for total PARP protein expression in SEM cells wildtype or deficient for IKZF1 exposed to prednisolone in the presence or absence of AKT inhibitors. Differences were tested for significance using an ANOVA followed by Dunnett’s multiple comparisons test.

Supplementary Figure 3
**(A)** Schematic overview representing the workflow used to analyze protein expression of wild-type (WT) and Ikzf1+/- mouse splenic B cells after *ex-vivo* treatment with prednisolone. Splenocytes were isolated and pre-stimulated by lipopolysaccharide (LPS) for 48 hours, whereafter viable cells were enriched by a Ficoll gradient. Cells were then incubated for 48h with 1 μg/ml prednisolone and protein samples were taken for immunoblot analysis. **(B)** Immunoblot analysis of AKT and pAKT protein expression levels of LPS-activated B cells obtained from 4 mice (2 wildtype versus 2 *Ikzf1*+/- mice). Actin was used as a loading control. **(C)** Immunoblot analysis of protein expression in SEM wt and SEM *IKZF1* ^-/-^ cells after a 3d treatment of 1 μM MK2206.

Supplementary Figure 4 Quantification of phospho-ERK, normalized for total ERK protein expression in SEM cells wildtype or deficient for IKZF1. The western blot results for 3 independent experiments were quantified, normalized and plotted. Differences were tested for significance using a student’s t-test

Supplementary Figure 5 Fold change in cell viability (AUC) upon prednisolone treatment relative to iberdomide-non-treated cells. t(1;19) rearranged PDXs, wt for IKZF1, were treated with indicated concentrations iberdomide after which responses to prednisolone were determined by quantification of cells positive for amine-reactive dyes using flow cytometry.

Supplementary Figure 6 Cell death as determined by quantification of cells positive for amine-reactive dyes using flow cytometry in ALL-PDXs carrying a heterozygous deletion of *IKZF1* after a 3-day treatment with increasing concentrations of prednisolone in the presence or absence of 1 μM MK2206, SCH772984 or their combination.
