# Supplementary figures and images for: Reversal of IKZF1-induced glucocorticoid resistance by dual targeting of AKT and ERK signaling pathways
Source: Front Oncol. 2022 Sep 2;12:905665. doi: 10.3389/fonc.2022.905665 (PMC9478899; doi:10.3389/fonc.2022.905665)

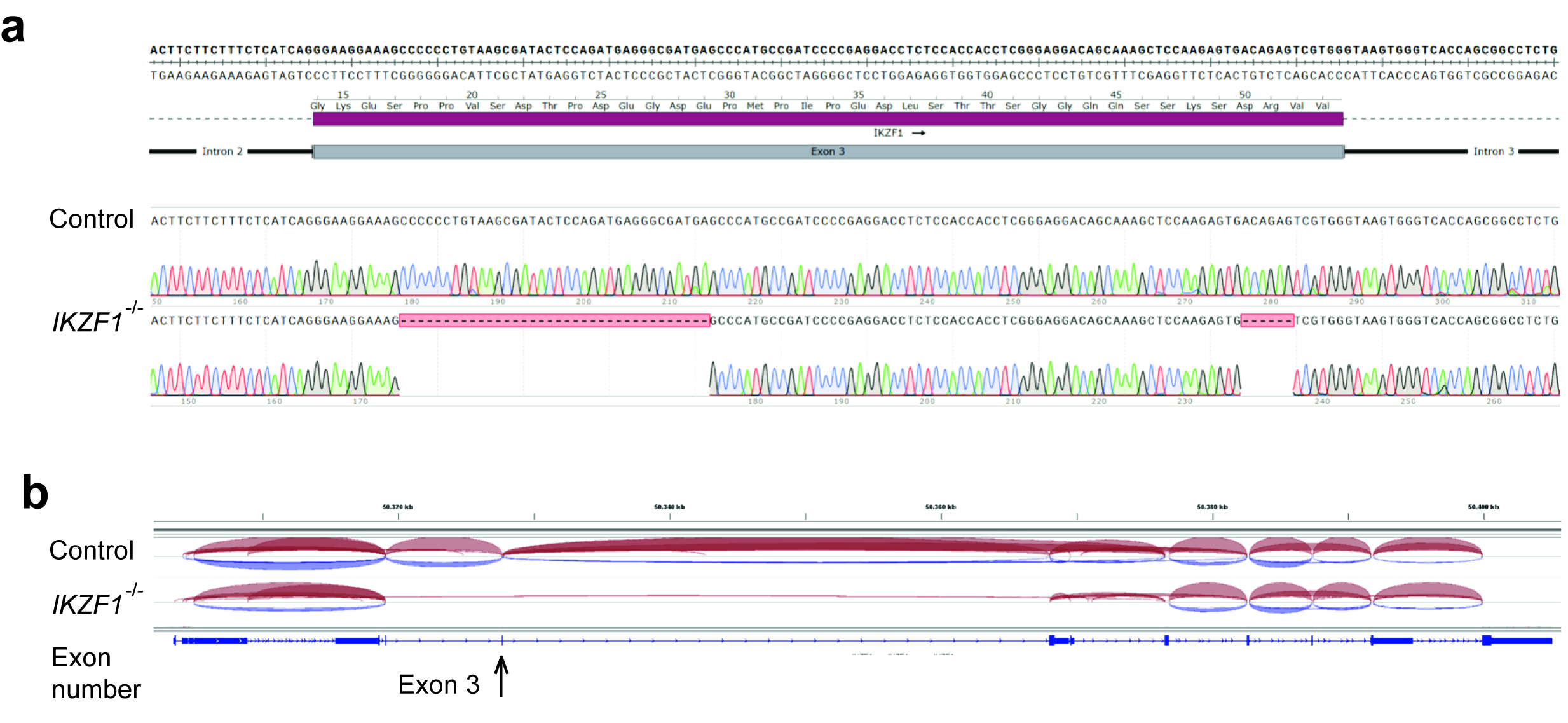

Supplement: Supplementary Figure 1 — (A) CRISPR/Cas9 was used to target the exon 3 locus of the IKZF1 gene in the SEM cells. After clonal expansion, the genomic DNA was analyzed by Sanger sequencing, revealing two insertions: c.del 51_86 CCCCCCTGTAAGCGATACTCC-AGATGAGGGCGATGA and c.del 149_154 ACAGAG (B) Analysis of mRNA sequencing data revealed that the genomic deletions resulted aberrant mRNA mRNA splicing and as a consequence, absence of detectable protein (). [file Image_1.tif]

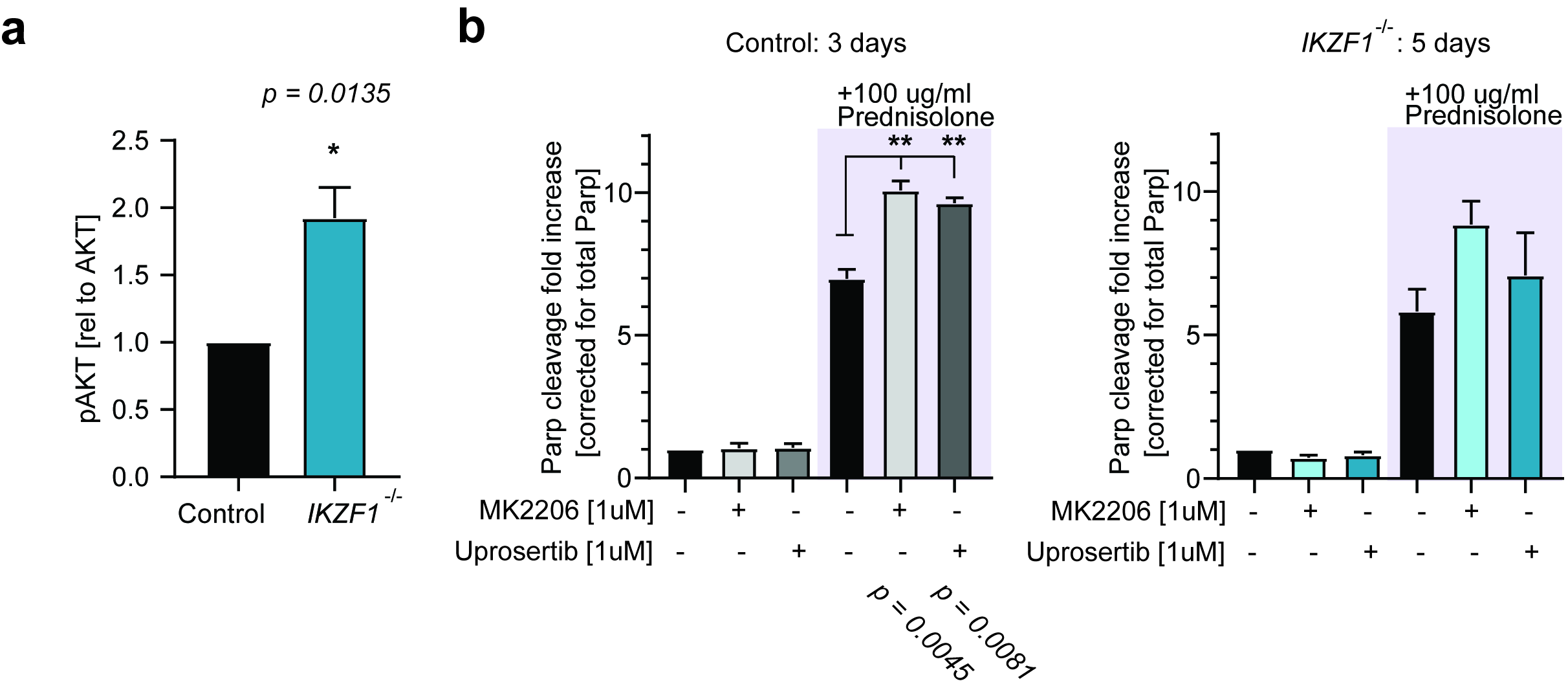

Supplement: Supplementary Figure 2 — (A) Quantification of phospho-AKT, normalized for total AKT protein expression in SEM cells wildtype or deficient for IKZF1. The western blot results for 3 independent experiments were quantified, normalized and plotted. Differences were tested for significance using a student’s t-test (B) Quantification of cleaved PARP protein, normalized for total PARP protein expression in SEM cells wildtype or deficient for IKZF1 exposed to prednisolone in the presence or absence of AKT inhibitors. Differences were tested for significance using an ANOVA followed by Dunnett’s multiple comparisons test. [file Image_2.tif]

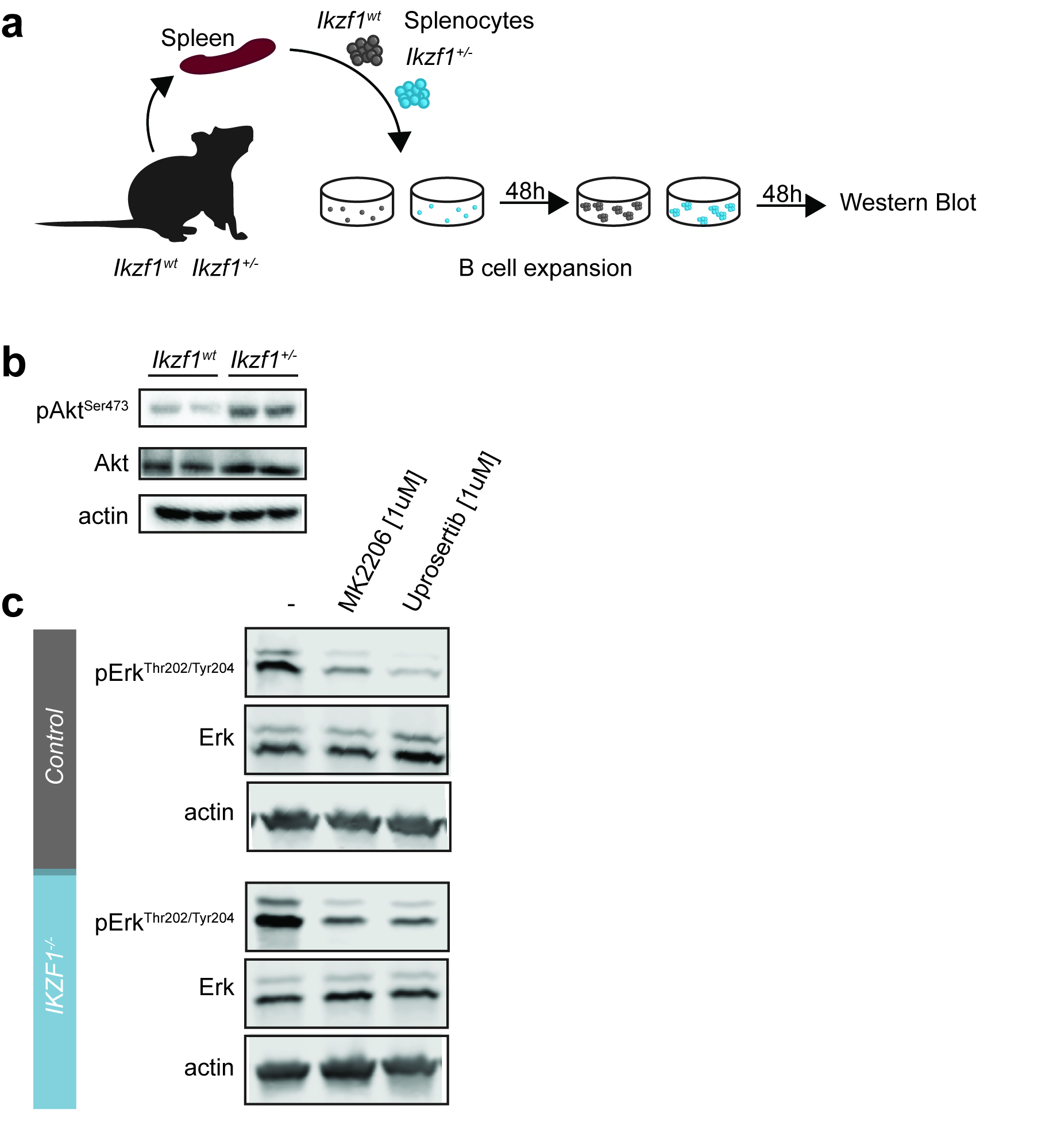

Supplement: Supplementary Figure 3 — (A) Schematic overview representing the workflow used to analyze protein expression of wild-type (WT) and Ikzf1+/- mouse splenic B cells after ex-vivo treatment with prednisolone. Splenocytes were isolated and pre-stimulated by lipopolysaccharide (LPS) for 48 hours, whereafter viable cells were enriched by a Ficoll gradient. Cells were then incubated for 48h with 1 μg/ml prednisolone and protein samples were taken for immunoblot analysis. (B) Immunoblot analysis of AKT and pAKT protein expression levels of LPS-activated B cells obtained from 4 mice (2 wildtype versus 2 Ikzf1+/- mice). Actin was used as a loading control. (C) Immunoblot analysis of protein expression in SEM wt and SEM IKZF1 -/- cells after a 3d treatment of 1 μM MK2206. [file Image_3.tif]

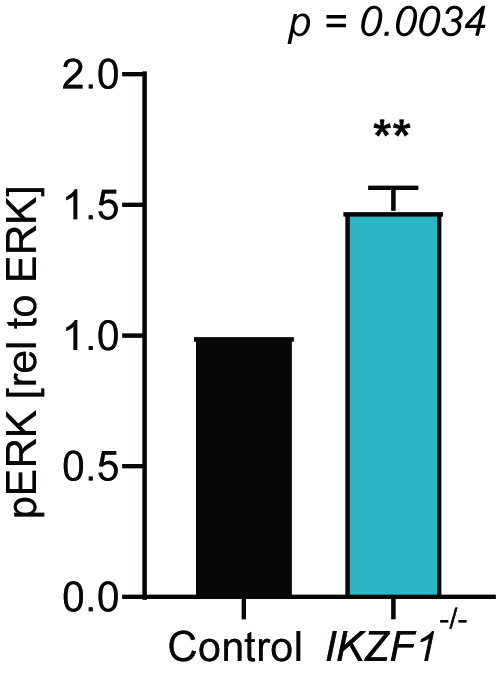

Supplement: Supplementary Figure 4 — Quantification of phospho-ERK, normalized for total ERK protein expression in SEM cells wildtype or deficient for IKZF1. The western blot results for 3 independent experiments were quantified, normalized and plotted. Differences were tested for significance using a student’s t-test [file Image_4.tif]

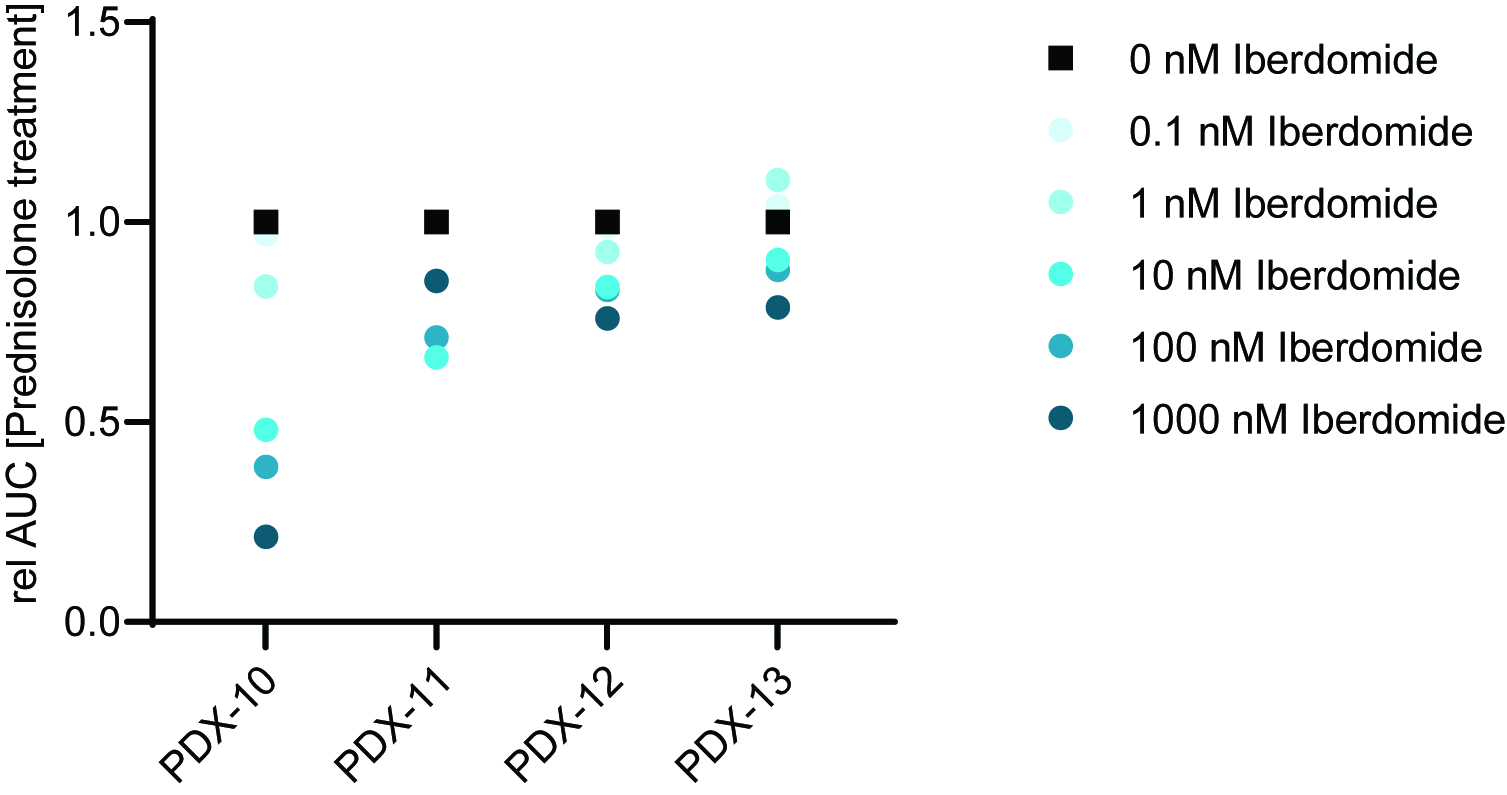

Supplement: Supplementary Figure 5 — Fold change in cell viability (AUC) upon prednisolone treatment relative to iberdomide-non-treated cells. t(1;19) rearranged PDXs, wt for IKZF1, were treated with indicated concentrations iberdomide after which responses to prednisolone were determined by quantification of cells positive for amine-reactive dyes using flow cytometry. [file Image_5.tif]

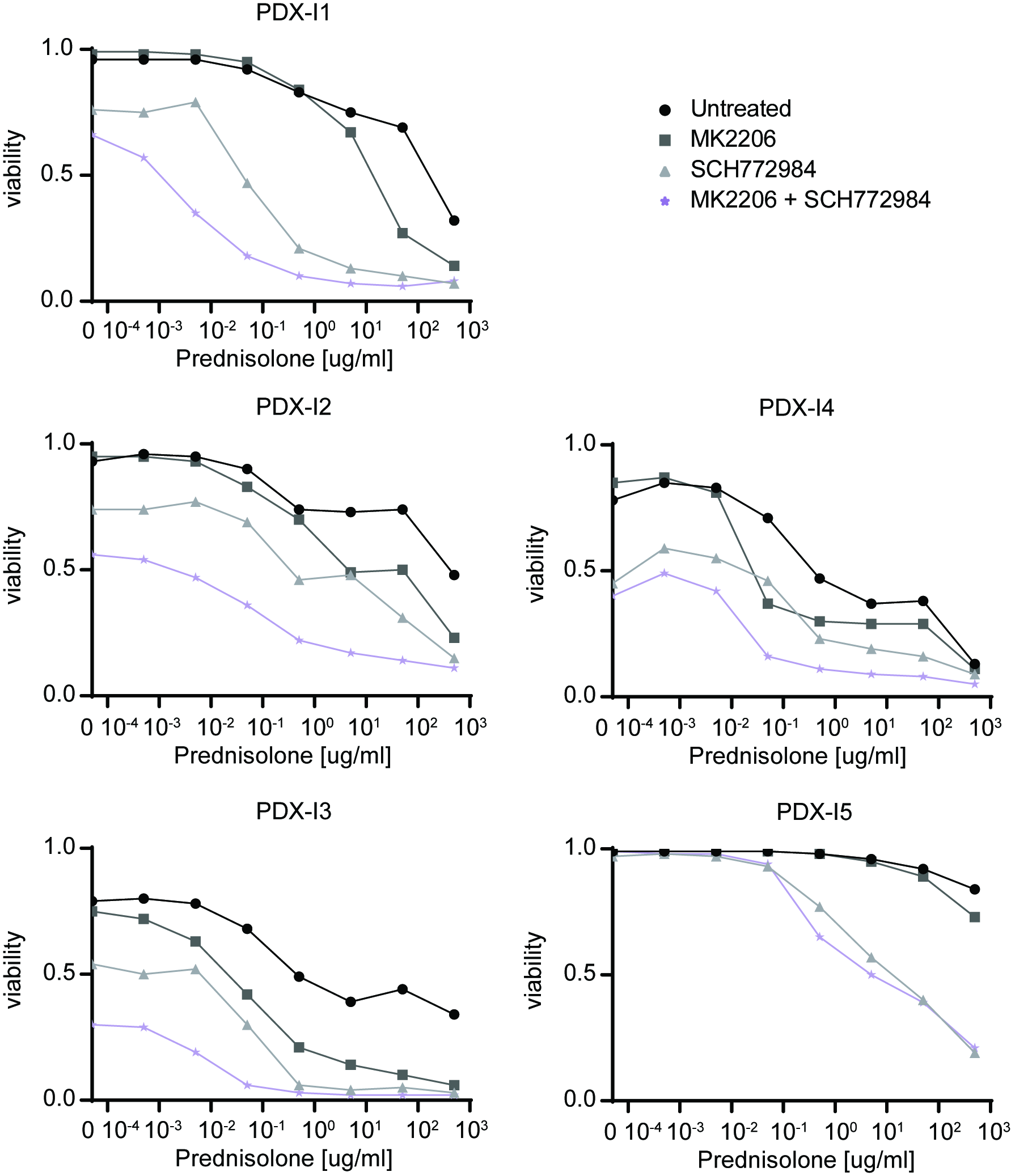

Supplement: Supplementary Figure 6 — Cell death as determined by quantification of cells positive for amine-reactive dyes using flow cytometry in ALL-PDXs carrying a heterozygous deletion of IKZF1 after a 3-day treatment with increasing concentrations of prednisolone in the presence or absence of 1 μM MK2206, SCH772984 or their combination. [file Image_6.tif]
