# Supplementary material for: Reversal of IKZF1-induced glucocorticoid resistance by dual targeting of AKT and ERK signaling pathways
Source: Front Oncol. 2022 Sep 2;12:905665. doi: 10.3389/fonc.2022.905665 (PMC9478899; doi:10.3389/fonc.2022.905665)
Supplement: Supplementary file 7 [file DataSheet_1.docx]

**Supplementary Table 1: Primer sequences**

| **Target** | **Application** | **Name** | **Sequence** |
| --- | --- | --- | --- |
| IKZF1 | gRNA | gRNA_IKZF1_Exon3.1_fw | TCATCTGGAGTATCGCTTAC |
|  |  | gRNA_IKZF1_Exon3.1_rv | GTAAGCGATACTCCAGATGA |
|  |  | gRNA_IKZF1_Exon3.3_fw | CTCCAAGAGTGACAGAGTCG |
|  |  | gRNA_IKZF1_Exon3.3_rv | CGACTCTGTCACTCTTGGAG |
|  | PCR | FW primer exon 3 | GTTCTCTCATTTGTATTGTGTGGG |
|  |  | RV primer exon 3 | GTTCTAACCCCAAACACTGAAAAG |
| TBP | RT-qPCR | FW | GCACAGGAGCCAAGAGTGAA |
|  |  | RV | ACATCACAGCTCCCCACCAT |
| TSC22D3 | RT-qPCR | FW | CCAGCGTGGTGGCCATAGAC |
|  |  | RV | GGATCTGCTCCTTCAGGATCTCCA |
| SGK1 | RT-qPCR | FW | ACCTTCTGTGGCACGCCGGA |
|  |  | RV | CTGTGTTTCGGCTATAAAAAGGC |
| BIM | RT-qPCR | FW | GCGCCAGAGATATGGAT |
|  |  | RV | CGCAAAGAACCTGTCAAT |
| ZFP36L2 | RT-qPCR | FW | TCGACCACACTTCTGTCCGCCTTC |
|  |  | RV | CACCGCCTTCTTGTCCAGCATGTT |
| NR3C1 | RT-qPCR | FW | CTGGGGACTCTGAACTTCCCTG |
|  |  | RV | CTGTTGTTGCTGTTGAGGAGCTGG |

**Supplementary Table 2 Characteristics of ALL-PDXs**

| **Patient number** | **Cytogenetics** |
| --- | --- |
| PDX-1 | MLLr |
| PDX-2 | n.a. |
| PDX-3 | E2A-HLF_t(17;19) |
| PDX-4 | n.a. |
| PDX-5 | hyperdiploid |
| PDX-6 | hypodiploid, deletion of TP53 |
| PDX-7 | TP53 del |
| PDX-8 | TYK2 mutation |
| PDX-9 | B-other |
| PDX-10 | E2A-PBX1_ T(1;19) PAX5/CDKN2A |
| PDX-11 | E2A-PBX1_ T(1;19) |
| PDX-12 | E2A-PBX1_ T(1;19) |
| PDX-13 | E2A-PBX1_ T(1;19) |
| PDX-I1 | EBF1del1-16, PAX5del2-8, ETV6del1-2 IL3del, CSF2RA, Ikzf1del4-7 |
| PDX-I2 | Gain, CRLF2, ETV6, Ikzf1del2-3 |
| PDX-I3 | Ikzf1del4-7, CDKN2ADel |
| PDX-I4 | Ikzf1del |
| PDX-I5 | ABL+, CDKN2A/BDel,PAX5Del,Ikzf1del1-8 P53Del |

**Supplementary Table 3: Antibodies and staining conditions**

| **Antibody** | **Supplier** | **Cat. number** | **Solvent** | **Dilution** |
| --- | --- | --- | --- | --- |
| actin | Sigma Aldrich | A4700 | 1% milk in TBS | 1:10000 |
| Ikaros | Santa Cruz | sc-398265 | 1% milk in TBS | 1:1000 |
| Ikaros | R&D systems | AF4984 | 5% BSA in TBS | 1:2000 |
| PARP | Cell Signaling Technologies | 9542 | 1% milk in TBS | 1:4000 |
| tubulin | Genetex | GTX628802 | 1% milk in TBS | 1:10000 |
| pAKT | Cell Signaling Technologies | 4060 | 5% BSA in TBS | 1:2000 |
| AKT | Cell Signalling Technology | 9272 | 1% milk in TBS | 1:2000 |
| pERK | Cell Signalling Technology | 4377 | 5% BSA in TBS | 1:2000 |
| ERK | Cell Signalling Technology | 4695 | 1% milk in TBS | 1:2000 |

**Supplementary Table 4: Statistical testing of differences in**

**prednisolone dose-response following AKT inhibition plotted in Figure 1b**

| **Statistical testing: AUC and one-way ANOVA** | | | | |
| --- | --- | --- | --- | --- |
| **Genotype** | **Treatment** | **AUC** | **Standard error** | **Significance** |
| Control | - | 168.9 | 7.697 | *** p=0.0002 |
|  | MK2206 | 87.83 | 4.891 |  |
|  | Uprostertib | 95.27 | 10.26 |  |
| IKZF1^-/-^ | - | 321.1 | 15.18 | **** p<0.0001 |
|  | MK2206 | 211.3 | 9.413 |  |
|  | Uprostertib | 196.8 | 5.820 |  |
| **Post-hoc statistical tests: Tukey’s multiple comparisons test** | | | | |
| **Genotype** | **Treatment** | | **p Value** | **Significance** |
| Control | - vs MK2206 | | 0.0002 | *** |
|  | - vs Uprosertib | | 0.0004 | *** |
|  | MK2206 vs Uprosertib | | 0.55 | ns |
| IKZF1^-/-^ | - vs MK2206 | | <0.0001 | **** |
|  | - vs Uprosertib | | <0.0001 | **** |
|  | MK2206 vs Uprosertib | | 0.26 | Ns |

**Supplementary Table 5: Statistical testing of differences in mRNA expression following prednisolone and/or AKT inhibitor treatment plotted in Figure 1D**

| **Genotype comparisons control vs IKZF1^-/-^ Statistical testing: student’s t-test** | | | | | | | |
| --- | --- | --- | --- | --- | --- | --- | --- |
| **Gene** | | **Treatment** | | **p value** | | **Significance** | |
| TSC22D3 | | Untreated | | 0.38 | | ns | |
|  |  | MK2206 | | 0.85 | | ns | |
|  |  | Uprosertib | | 0.56 | | ns | |
|  |  | Prednisolone | | 0.0006 | | *** | |
|  |  | MK2206 + Prednisolone | | 0.14 | | ns | |
|  |  | Uprosertib + Prednisolone | | 0.80 | | ns | |
| ZFP36L2 | | Untreated | | 0.095 | | ns | |
|  |  | MK2206 | | 0.074 | | ns | |
|  |  | Uprosertib | | 0.016 | | * | |
|  |  | Prednisolone | | 0.61 | | ns | |
|  |  | MK2206 + Prednisolone | | 0.13 | | ns | |
|  |  | Uprosertib + Prednisolone | | 0.78 | | ns | |
| BIM | | Untreated | | 0.71 | | ns | |
|  |  | MK2206 | | 0.66 | | ns | |
|  |  | Uprosertib | | 0.55 | | ns | |
|  |  | Prednisolone | | 0.0041 | | ** | |
|  |  | MK2206 + Prednisolone | | 0.054 | | ns | |
|  |  | Uprosertib + Prednisolone | | 0.39 | | ns | |
| SGK1 | | Untreated | | 0.11 | | ns | |
|  |  | MK2206 | | 0.16 | | ns | |
|  |  | Uprosertib | | 0.097 | | ns | |
|  |  | Prednisolone | | 0.074 | | ns | |
|  |  | MK2206 + Prednisolone | | 0.20 | | ns | |
|  |  | Uprosertib + Prednisolone | | 0.84 | | ns | |
| NR3C1 | | Untreated | | 0.94 | | ns | |
|  |  | MK2206 | | 0.58 | | ns | |
|  |  | Uprosertib | | 0.54 | | ns | |
|  |  | Prednisolone | | 0.36 | | ns | |
|  |  | MK2206 + Prednisolone | | 0.14 | | ns | |
|  |  | Uprosertib + Prednisolone | | 0.044 | | * | |
|  | | | | | | | |
| **Differences upon treatment Statistical testing: one-way ANOVA with post-hoc Dunnett’s multiple comparisons test** | | | | | | | |
| **Gene** | **Genotype** | | **Treatment** | | **p value** | | **Significance** |
| TSC22D3 | Control | | Untreated vs MK2206 | | 0.027 | | * |
|  |  |  | Untreated vs Uprosertib | | 0.018 | | * |
|  |  |  | Prednisolone vs MK + Pred | | 0.0013 | | ** |
|  |  |  | Prednisolone vs Upro + Pred | | 0.0053 | | ** |
|  | IKZF1^-/-^ | | Untreated vs MK2206 | | 0.11 | | ns |
|  |  |  | Untreated vs Uprosertib | | 0.25 | | ns |
|  |  |  | Prednisolone vs MK + Pred | | 0.0004 | | *** |
|  |  |  | Prednisolone vs Upro + Pred | | 0.0004 | | *** |
| ZFP36L2 | Control | | Untreated vs MK2206 | | 0.0044 | | ** |
|  |  |  | Untreated vs Uprosertib | | 0.0072 | | ** |
|  |  |  | Prednisolone vs MK + Pred | | 0.0007 | | *** |
|  |  |  | Prednisolone vs Upro + Pred | | 0.0039 | | ** |
|  | IKZF1^-/-^ | | Untreated vs MK2206 | | 0.0083 | | ** |
|  |  |  | Untreated vs Uprosertib | | 0.070 | | ns |
|  |  |  | Prednisolone vs MK + Pred | | 0.14 | | ns |
|  |  |  | Prednisolone vs Upro + Pred | | 0.088 | | ns |
| BIM | Control | | Untreated vs MK2206 | | 0.12 | | ns |
|  |  |  | Untreated vs Uprosertib | | 0.097 | | ns |
|  |  |  | Prednisolone vs MK + Pred | | 0.086 | | ns |
|  |  |  | Prednisolone vs Upro + Pred | | 0.27 | | ns |
|  | IKZF1^-/-^ | | Untreated vs MK2206 | | 0.20 | | ns |
|  |  |  | Untreated vs Uprosertib | | 0.26 | | ns |
|  |  |  | Prednisolone vs MK + Pred | | 0.0003 | | *** |
|  |  |  | Prednisolone vs Upro + Pred | | 0.0006 | | *** |
| SGK1 | Control | | Untreated vs MK2206 | | 0.32 | | ns |
|  |  |  | Untreated vs Uprosertib | | 0.33 | | ns |
|  |  |  | Prednisolone vs MK + Pred | | 0.99 | | ns |
|  |  |  | Prednisolone vs Upro + Pred | | 0.34 | | ns |
|  | IKZF1^-/-^ | | Untreated vs MK2206 | | 0.26 | | ns |
|  |  |  | Untreated vs Uprosertib | | 0.34 | | ns |
|  |  |  | Prednisolone vs MK + Pred | | 0.032 | | * |
|  |  |  | Prednisolone vs Upro + Pred | | 0.10 | | ns |
| NR3C1 | Control | | Untreated vs MK2206 | | 0.012 | | * |
|  |  |  | Untreated vs Uprosertib | | 0.065 | | ns |
|  |  |  | Prednisolone vs MK + Pred | | 0.021 | | * |
|  |  |  | Prednisolone vs Upro + Pred | | 0.037 | | * |
|  | IKZF1^-/-^ | | Untreated vs MK2206 | | 0.45 | | ns |
|  |  |  | Untreated vs Uprosertib | | 0.78 | | ns |
|  |  |  | Prednisolone vs MK + Pred | | 0.17 | | ns |
|  |  |  | Prednisolone vs Upro + Pred | | 0.57 | | ns |

**Supplementary Table 6: Statistical testing of differences in prednisolone dose-response following AKT and or ERK inhibition plotted in Figure 2b**

| **Statistical testing: AUC and One way-ANOVA** | | | | |
| --- | --- | --- | --- | --- |
| Genotype | treatment | AUC | Standard error | Significance |
| Control | - | 157.7 | 21.68 | **** p=0<0001 |
|  | MK2206 | 72.04 | 2.97 |  |
|  | SCH772984 | 44.64 | 4.53 |  |
|  | MK2206 + SCH772984 | 32.32 | 0.69 |  |
| IKZF1^-/-^ | - | 317.9 | 27.26 | **** p<0.0001 |
|  | MK2206 | 208.3 | 16.28 |  |
|  | SCH772984 | 117.5 | 14.20 |  |
|  | MK2206 + SCH772984 | 78.4 | 6.63 |  |
| **Post-hoc statistical tests: Tukey’s multiple comparisons test** | | | | |
| Genotype | treatment | | p Value | Significance |
| Control | - vs MK2206 | | <0.0001 | **** |
|  | - vs SCH772984 | | <0.0001 | **** |
|  | - vs MK2206 + SCH772984 | | <0.0001 | **** |
|  | MK2206 vs SCH772984 | | 0.059 | ns |
|  | MK2206 vs MK2206 + SCH772984 | | 0.010 | * |
|  | SCH772984 vs MK2206 + SCH772984 | | 0.60 | ns |
| IKZF1^-/-^ | - vs MK2206 | | 0.0003 | *** |
|  | - vs SCH772984 | | <0.0001 | **** |
|  | - vs MK2206 + SCH772984 | | <0.0001 | **** |
|  | MK2206 vs SCH772984 | | 0.0010 | ** |
|  | MK2206 vs MK2206 + SCH772984 | | <0.0001 | **** |
|  | SCH772984 vs MK2206 + SCH772984 | | 0.10 | ns |
